# Final Demonstration of the Co-Identity of Lipiarmycin A3 and Tiacumicin B (Fidaxomicin) through Single Crystal X-ray Analysis

**DOI:** 10.3390/antibiotics6010007

**Published:** 2017-02-08

**Authors:** Stefano Serra, Luciana Malpezzi, Angelo Bedeschi, Claudio Fuganti, Piera Fonte

**Affiliations:** 1 Consiglio Nazionale delle Ricerche (C.N.R.), Istituto di Chimica del Riconoscimento Molecolare, Via L. Mancinelli 7, I-20131 Milano, Italy; antibiotics@mdpi.com; 2 Dipartimento di Chimica, Materiali ed Ingegneria Chimica del Politecnico, Via Mancinelli 7, I-20131 Milano, Italy; luciana.malpezzi@polimi.it; 3 OLON S.p.A, Strada Rivoltana 6/7, 20090 Rodano, Italy; abedeschi@olonspa.it (A.B.); pfonte@olonspa.it (P.F.)

**Keywords:** actinomycetes, antibiotic, fidaxomicin, lipiarmycin A3, tiacumicin B, X-ray analysis

## Abstract

Lipiarmycin A3 and tiacumicin B possess the same chemical structure and have been considered identical till recently, when some authors have suggested the possibility of a minor difference between the chemical structures of the two antibiotics. In this work we performed a comparative X-ray analysis of lipiarmycin A3 and tiacumicin B. Although the commercial samples of the aforementioned compounds crystallize into two different crystal systems—evidently due to the different crystallization conditions—their chemical structures are identical. These results confirmed the previous assigned chemical structure of lipiarmycin A3 and its absolute configuration as well as its co-identity with the chemical structure of tiacumicin B, providing the definitive proof that these pharmaceutical compounds are identical in all respects.

## 1. Introduction

During the early 1970s, the Lepetit group (Italy) undertook a screening for antibiotics produced by strains of the genus *Actinoplanes*. A new *Actinoplanes* species [1] was isolated from a soil specimen collected in Decca in India, and accordingly, the bacteria was named *Actinoplanes deccanensis*. This microorganism was found to produce a substance with strong activity against gram-positive bacteria, including strains resistant to several antibiotics. The new antibiotic was isolated on 29 February 1972, and was named lipiarmycin (from leap year).

Although its chemical–physical properties, its biological activity [2,3], and its NMR characterization [4] were thoroughly described within a few years, this material was only recognized to be a mixture of two related products in 1987, when the first comprehensive study [5] on the chemical structure determination of lipiarmycin was published. Based on chemical degradations and NMR studies, the two antibiotics extracted from *Actinoplanes deccanensis*—named lipiarmycin A3 (**1**) and lipiarmycin A4 (**2**)—proved to be characterized by a common 18-membered macrolactone attached to two glycosyl moieties, namely 2-*O*-methyl-4-*O*-homodichloro-orsellinate-β-d-rhamnose and 4-*O*-isobutyrate-5-methyl-β-rhamnose.

**Figure 1:**
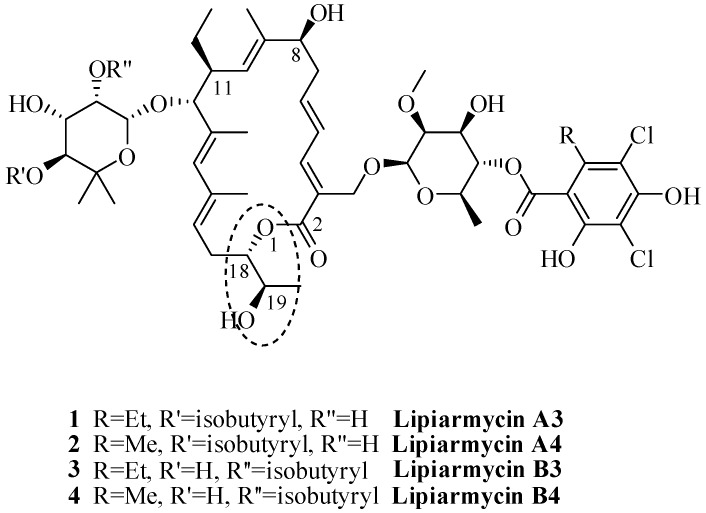
Chemical structures of lipiarmycins.

More specifically, the gross structural formulas (stereochemistry of the asymmetric centers of the lactone moiety not attributed) of **1** and **2** were assigned to lipiarmycin A3 and lipiarmycin A4, respectively (Figure 1).

Further studies [6] revealed that the same strain was also able to produce two minor compounds, lipiarmycins B3 (**3**) and B4 (**4**), respectively, which differ from the corresponding A3 and A4 by the position of the isobutyric ester on the rhamnose moiety.

In the meantime, two research groups from Kitasato University (Japan) and Abbot Laboratory (United States) independently set up screening for antibiotics produced by soil-isolated microorganisms, and reported the isolation of clostomicins from *Micromonospora echinospora* subsp. *Armeniaca* [7] and tiacumicins from *Dactylosporangium aurantiacum* subsp. *Hamdenensis* [8], respectively.

Based on NMR studies, clostomicin B1 and tiacumicin B were recognized to be identical to lipiarmycin A3, although the stereochemistry of the macrolactone moiety remained unassigned.

The increasing interest in tiacumicin B as the therapeutic agent of choice for the treatment of *Clostridium difficile* infection [9,10,11] induced more detailed structural studies on this class of compounds, culminating in the determination of the single crystal X-ray analysis of tiacumicin B [12,13]. The stereo structure **1**, possessing the (18*S*,19*R*) absolute configuration, was thus assessed for tiacumicin B. In this context [12], the 19-oxo analogue of tiacumicin B was obtained and reduced with NaBH_4_ in methanol. This process was expected to afford the (19*S*)-diastereoisomer of **1**. Curiously, the spectroscopic analyses of the latter compound were not compared to those of tiacumicin B, but appeared to be identical to those reported for lipiarmycin A4. However, the NMR spectra of the semi-synthetic and of the tiacumicin B (of unambiguously determined configuration) were acquired in different solvents, thus inhibiting the evaluation of the subtle spectroscopic differences between the two. Following the same reasoning, to lipiarmycin A4 was assigned the (19*S*) configuration without any further confirmatory X-ray analysis.

According to the pharmaceutical regulations adopted by the majority of nations, even a small difference in the chemical structure of two pharmaceutical products implies the need for the registration of two different drugs. Therefore, the determination of the lipiarmycin A3 and tiacumicin B structures and/or the demonstration of their co-identity have become a relevant issue for the pharma industry [14]. In addition, recent studies [15] have demonstrated that lipiarmycin possesses good activity against multidrug-resistant strains of *Mycobacterium tuberculosis*, definitely expanding its pharmaceutical relevance.

**Figure 2:**
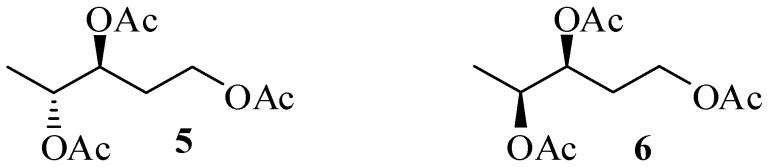
The pentane-1,3,4-triol derivatives used to assign the C18-C19 relative configuration to lipiarmycin A3 and tiacumicin B.

For that reason, we have recently reported a comprehensive study on this topic [16]. In our hands, samples of tiacumicin B from *Dactylosporangium auranticum* and lipiarmycin A3 from *Actinoplanes* showed absolutely superimposable proton and carbon spectra both in methanol and in chloroform, which strongly suggested that they have the same structure. The chemical degradation of each of the above-described samples allowed the isolation of the same derivative **5** (Figure 2) that proved to be identical to a synthetic sample of **5** and was clearly distinguishable by GC and NMR analysis from its diastereoisomer **6**. As a consequence, we unambiguously demonstrated that lipiarmycin A3 and tiacumicin B possess the same (18*S*,19*R*) absolute configuration, and thus they might be identical.

One year later, three different groups independently reported [17,18,19] the enantioselective synthesis of the tiacumicin B aglycon and of the putative lipiarmycin aglycon, followed by the first total synthesis of fidaxomicin [20]. The latter synthetic compound proved to be identical to a commercial sample of tiacumicin B from *Dactylosporangium auranticum*, thus confirming the structure assigned and substantiating our work on the lipiarmycin–tiacumicin co-identity.

Despite this fact, lipiarmycin’s structure has yet to be confirmed by X-ray analysis. Moreover, the analytic data of synthetic tiacumicin prepared by total synthesis have been compared only with those of the natural antibiotic extracted from *Dactylosporangium auranticum*. Consequently, we decided to perform a comparative X-ray analysis of two commercial samples of both lipiarmycin and tiacumicin. Here, we report the results of this study that unambiguously indicates that lipiarmycin and tiacumicin are the same compound in all respects.

## 2. Results and Discussion

Fidaxomicin (and more broadly most active pharmaceutical ingredients, APIs)—owing to the large dimension of the molecules, the presence of many functional centres, and the possibility of forming different hydrogen bonding in the crystal packing—can adopt different modes of self-assembly in the solid state, and can consequently exist as polymorphic, solvate, and/or hydrated forms.

Fidaxomicin in particular has been found to crystallize into two differently-solvated crystalline phases, arising from different crystallization procedures.

**Figure 3:**
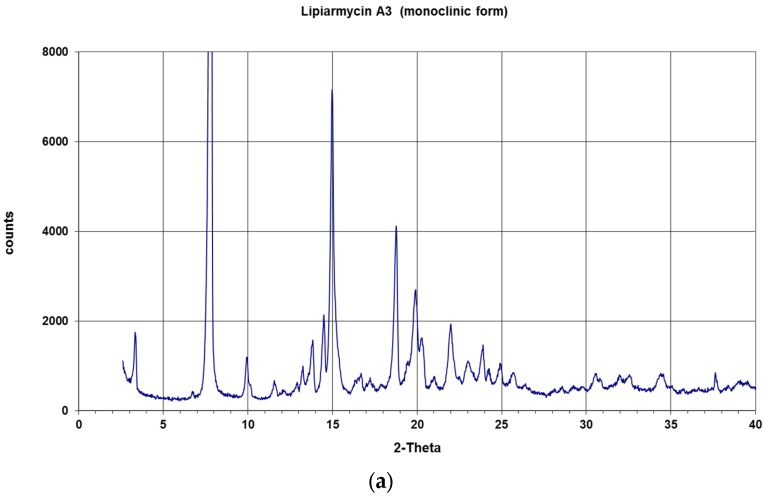

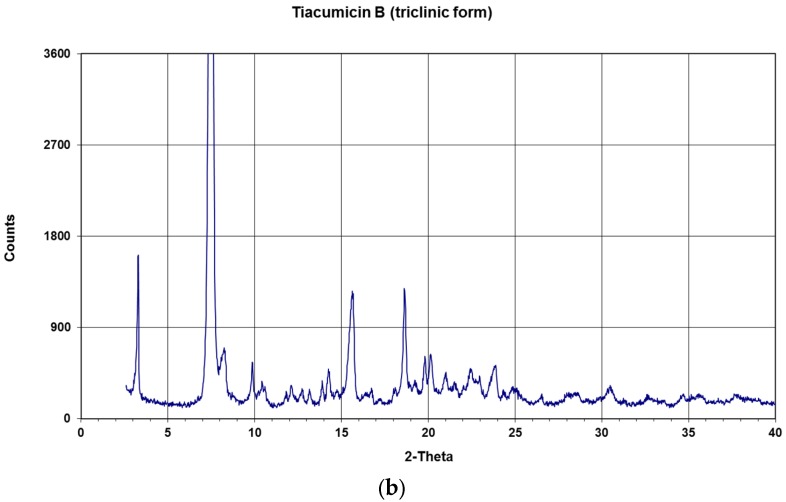
X-ray powder diffraction (XRPD) diagrams of crystalline fidaxomicin: (**a**) lipiarmycin A3 (monoclinic phase); (**b**) tiacumicin B (triclinic phase). For details of the two crystalline phases, see text.

The X-ray powder diffraction patterns of the two crystalline phases were collected on the freshly prepared samples, and are reported in Figure 3a,b.

**Figure 4:**
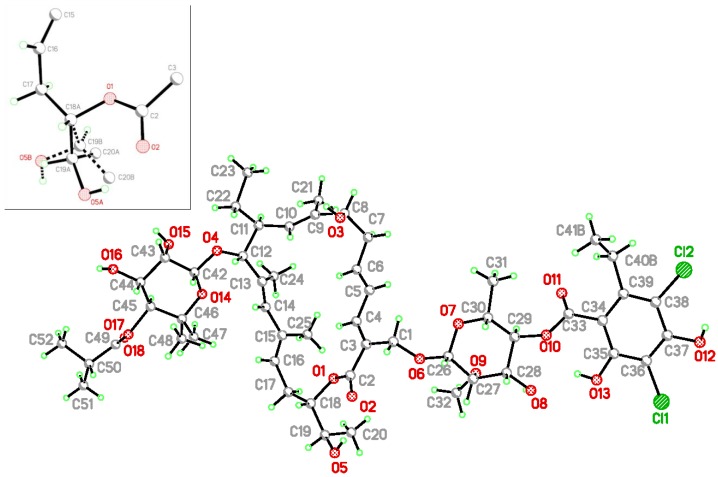
The molecular structure of lipiarmycin A3, showing the atomic labelling scheme. In the inset, a zoom is given of the two disordered positions of the C19 group, linked to C18 atom.

Lipiarmycin A3 crystallizes in the monoclinic system, space group P2_1_, with a partial disordered water molecule (site occupation factor of 0.8). The molecules show a high degree of disorder and/or thermal motion, as judged by large displacement parameters of many atoms: an attempt was made to split many atoms into two positions, the refinement of the structure was carried out up to convergence but the results appeared to be unsuitable: the discrepancy factor (R-factor) did not improved so significantly to justify the split of the most part of the molecule. The disorder included in the last refinement was limited to the most shaking external groups. Figure 4 displays the molecular structure of lipiarmycin A3 and shows in the inset a zoom onto the two disordered positions of the C19 group linked to C18 atom.

Tiacumicin B crystallizes in the triclinic system with two independent molecules (named mol A and mol B) in the unit cell and several disordered solvent molecules (water and methanol) that participate in the crystal packing. The presence of the solvent deforms the monoclinic cell of lipiarmycin A3 batch, giving rise to a triclinic cell having *a* axis slightly longer, and *b* and *c* axes nearly similar to those of a monoclinic cell. Moreover, some terminal groups of the molecules appeared disordered or affected by large thermal motion. Due to the distortion introduced by the presence of solvent molecules and by disorder, the two molecules of the monoclinic cell of lipiarmycin A3 sample are no longer symmetrically equivalent, and become the two independent molecules in the triclinic lattice of the tiacumicin B sample.

**Figure 5:**
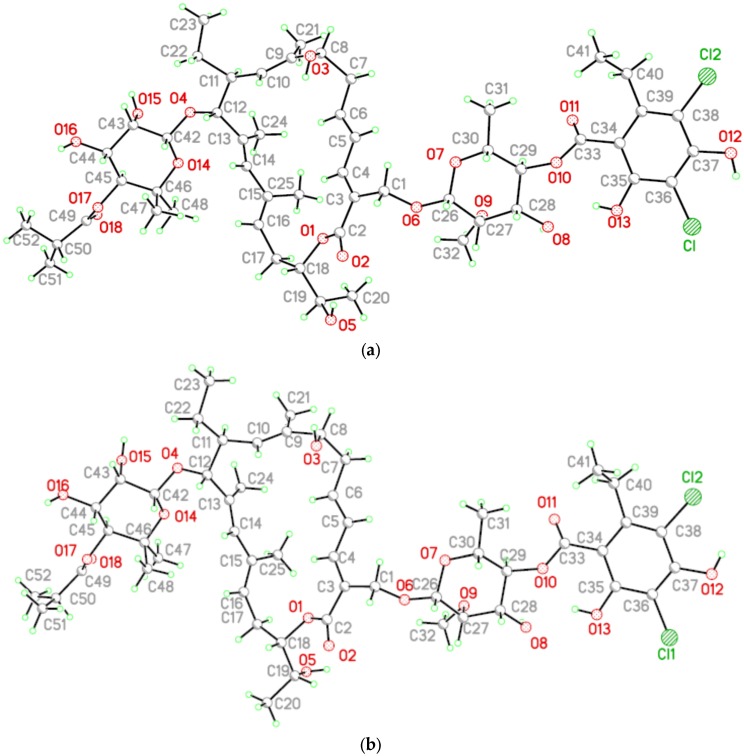
The molecular structure of tiacumicin B showing the two independent molecules in the unit cell with the atomic labelling schemes: (**a**) mol A and (**b**) mol B.

The geometry of the two molecules A and B were found to be very similar, the most relevant difference being the rotation angle about the C18–C19 bond (see Figure 5).

Despite the different crystalline system, the molecular conformation of the two samples are almost the same, as can be seen by comparison of Figure 4 and Figure 5.

This finding confirms that lipiarmycin A3 and tiacumicin B correspond to two different crystalline systems of the same substance.

It is worth noting that the same molecular geometry and conformation has also been found in the tiacumicin B acetone solvate [13]. This structure shows a monoclinic cell comparable with that of lipiarmycin, just with an expected lengthening of the *a* axis, owing to the presence of solvent molecule.

The absolute configurations of the molecules were unequivocally established based on Flack parameters, the values of which guarantee the correctness of the assignment.

The sequence of the absolute configuration at the 14 chiral centres for lipiarmycin A3 and tiacumicin B was found to be: 8*S*, 11*S*, 12*R*, 18*S*, 19*R*, 26*R*, 27*S*, 28*S*, 29*S*, 30*R*, 42*R*, 43*S*, 44*R*, 45*S*.

Obviously, the disorder affecting some part of the molecules does not alter the absolute configuration of the involved atoms in any way. It is relevant that the high degree of disorder and thermal motion in the crystal lattices of both samples were found, even though data collections were carried out at low temperature (100 K).

**Figure 6:**
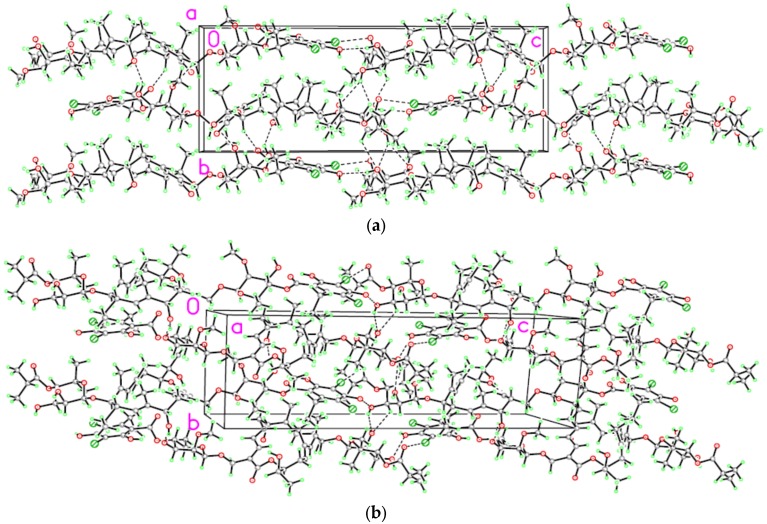
The molecular packing of the two fidaxomicin crystalline phases, viewed along the *a* axis: (**a**) monoclinic form; (**b**) triclinic form. Dashed lines indicate intermolecular halogen and hydrogen bonds.

The packing of the molecules in the crystal lattices are similar for monoclinic and triclinic forms. The packing diagrams for both samples (see Figure 6a,b), viewed along the *a* direction, show the formation of chains of molecules joined head-to-tail by hydrogen bonding of type O15-H…O12 and by halogen bonding of type C36-Cl1…O18 (Table 1).

Additional lateral hydrogen bonding linking molecules of adjacent chains gives rise to a supramolecular three-dimensional structure.

The difference in the lattice angles between triclinic and monoclinic cells causes a different orientation of the chains with respect to the crystal lattice: in the triclinic form, the chains run parallel to the [001] direction, while in the monoclinic form, the chains extend along the [101] direction.

In the triclinic form, the distribution of molecules in the lattice forms a continuous solvent accessible channel parallel to the *b* axis giving rise to a small lengthening of the *a* axis. Some water and methanol molecules arrange themselves in a disordered arrangement within the channel.

## 3. Materials and Methods

### 3.1. Materials

Lipiarmycin A3 (**1**): was extracted from *Actinoplanes deccanensis*, ATCC 21983, and was supplied by Olon S.p.A. and crystallized from water/methanol.

Tiacumicin B (**1**): from *Dactylosporangium auranticum*, subsp. *Hamdenensis,* NRRL 18085, was supplied by Olon S.p.A. and crystallized from water/methanol.

### 3.2. Single Crystal Structure Determination

Crystal data for the monoclinic form: C_52_H_74_O_18_Cl_2_·0.8H_2_O, M_r_ = 1070.8, light yellow crystal of size 0.40 × 0.14 × 0.10 mm, monoclinic system, P2_1_ space group, *a* = 11.5020(3) Å, *b* = 9.2400(3) Å, *c* = 26.2524(82) Å, β = 99.037(2)°, V = 2755.4(1) Å^3^, Z = 2, D_c_ = 1.291 g·cm^−3^, μ = 0.190 mm^−1^, F(000) = 1141; T = 103 K. A total of 104,514 reflections (independent 16,154, (R(int) = 0.041)) collected. The final stage of refinement, for 810 parameters refined and 36 restraints, converged to R_1_ = 0.0585 (R_w_ = 0.1228) for 12,008 observed reflections with I ≥ 2σ(I). The molecules show disorder and high degree of thermal motion. A partial disordered molecule of water was found in the unit cell; the goodness of fit, S, was 1.041. The final difference map showed a maximum and minimum residual peak of 0.921 and −1.056 eÅ^−3^, respectively. Flack parameter [21] led to a value of 0.02(5) for the selected absolute configuration and to a value of 0.99(5) for the opposite one.

The pertinent CIF file has been deposited at Cambridge Crystallographic Data Centre under deposition code 1481503.

Crystal data for triclinic form: 2(C_52_H_74_O_18_Cl_2_)·3.2(CH_3_ OH)·2.8(H_2_O), M_r_ = 1127.01, colourless crystal of size 0.43 × 0.18 × 0.12 mm, found after several attempts to obtain a crystal suitable enough for single crystal X-ray diffraction , triclinic system, P1 space group, *a* = 12.5814(9) Å, *b* = 9.1840(7) Å, *c* = 26.774(2) Å, α = 88.776(4)°, β = 89.069(4)°, γ = 106.326(4)°, V = 2968.0(4) Å^3^, Z = 2, D_c_ = 1.259 g·cm^−3^, μ = 0.182 mm^−1^, F(000) = 1195; T = 103 K. A total of 57,770 reflections (independent 19,975, [R(int) = 0.033]) collected. Two independent molecules and several solvent molecules in the unit cell. Due to the high number of atoms in the structure, the two independent molecules were refined separately. The final stage of refinement, with 1477 parameters refined and 112 restraints, converged to R = 0.0592 (R_w_ = 0.1522) for 17,666 observed reflections having I ≥ 2σ(I). The goodness of fit, S, was 1.055. The final difference map showed a maximum and minimum residual peak of 0.599 and −0.389 eÅ^−3^, respectively. Flack parameter = 0.08(5) for the selected absolute configuration and 0.93(5) for the opposite one.

The pertinent CIF file has been deposited at Cambridge Crystallographic Data Centre under deposition code 1481504.

For both structures: data collection was carried out on a SMART-APEX CCD area detector diffractometer by using a graphite monochromated Mo-Kα radiation (λ = 0.71073 Å). Empirical adsorption correction was applied using the SADABS program.

The structures were solved by direct methods using SIR-97 [22], the refinement were carried out on F^2^ by full-matrix least-squares procedure with SHELXL97 [23], with anisotropic temperature factors for non-H atoms. H atoms were positioned geometrically and refined in a riding model. The extended disorder and the thermal motion found in both structures and the large number of atoms to be refined in the triclinic form could account for the presence of same alerts of level A and B in the CIFCHECK output of the last refinement.

Owing to the presence of chlorine atoms in the molecules, the correct absolute configurations were unambiguously established by refinement of the Flack parameter [21].

## 4. Conclusions

Although lipiarmycin A3 and tiacumicin B crystallize into two different crystal systems—evidently due to the different crystallization conditions—the chemical structures of the two compounds are identical (Figure 3a). The results presented in our work unambiguously demonstrated this fact. The determined crystal structures confirmed the previous assigned chemical structure of lipiarmycin A3 and its absolute configuration, as well as its co-identity with the chemical structure of tiacumicin B. Indeed, owing to the presence of heavy chlorine atoms in the molecules, the absolute configurations of both crystals have been unambiguously assigned based on the Flack parameter. In conclusion, the comparative X-ray analysis of lipiarmycin A3 and tiacumicin B provides the definitive proof that these pharmaceutical compounds are identical in all respects.

## Figures and Tables

**Table 1:** Halogen- and hydrogen-bond data [Å ,°] for monoclinic form (I) and triclinic form (II).

**C−Cl···O**	**C−Cl**	**Cl···O**	**C−Cl···O**	
(I)				
C36−Cl1···O18 ^i^	1.724(3)	3.233(4)	170.0(1)	
(II)				
C36A−Cl1A···O18A ^ii^	1.743(4)	3.158(4)(4)	161.8(2)	
C36B−Cl1B···O18B ^i^	1.723(5)	3.033(9)	162.4(3)	
**D−H...A**	**d(D−H)**	**d(H···A)**	**d(D···A)**	**D−H···A**
(I)				
O8−H8O···O9	0.84	2.28	2.719(2)	112.9
O13-H13O···O10	0.84	2.10	2.736(2)	132.5
O13-H13O···O8	0.84	2.18	2.907(2)	144.5
O15-H15O···O12 ^iii^	0.84	2.20	2.883(2)	138.7
O16-H16O···O15 ^iv^	0.84	2.24	2.954(3)	143.6
O16-H16O···O14 ^iv^	0.84	2.49	3.211(3)	144.7
(II)				
O3A-H3OA...O8B ^v^	0.84	2.187	2.754(3)	124.8
O8A-H8OA...O3B ^ii^	0.84	2.166	2.831(3)	136.0
O12A-H12O...O16A ^ii^	0.84	2.037	2.752(4)	142.6
O13A-H13O...O8A	0.84	2.040	2.773(4)	145.4
O16A-H16O...O15B ^vi^	0.84	2.170	2.940(3)	154.0
O3B-H3OB...O5B ^vii^	0.84	2.187	2.754(3)	124.8
O8B-H8OB...O3A ^viii^	0.84	1.950	2.769(4)	164.6
O13B-H13O...O8B	0.84	2.087	2.809(4)	143.7

Symmetry code: ^i^ x, y, z−1; ^ii^ x, y, −z + 1 ; ^iii^ x + 1, y, z + 1; ^iv^ −x, y + 1/2, −z+1; ^v^ x, y, z + 1; ^vi^ x, y + 1, z; ^vii^ x−1, y, z; ^viii^ x, y−1, z−1.
